# Markers of Polyfunctional SARS-CoV-2 Antibodies in Convalescent Plasma

**DOI:** 10.1128/mBio.00765-21

**Published:** 2021-04-20

**Authors:** Harini Natarajan, Andrew R. Crowley, Savannah E. Butler, Shiwei Xu, Joshua A. Weiner, Evan M. Bloch, Kirsten Littlefield, Wendy Wieland-Alter, Ruth I. Connor, Peter F. Wright, Sarah E. Benner, Tania S. Bonny, Oliver Laeyendecker, David Sullivan, Shmuel Shoham, Thomas C. Quinn, H. Benjamin Larman, Arturo Casadevall, Andrew Pekosz, Andrew D. Redd, Aaron A. R. Tobian, Margaret E. Ackerman

**Affiliations:** a Department of Microbiology and Immunology, Geisel School of Medicine at Dartmouth, Dartmouth College, Hanover, New Hampshire, USA; b Thayer School of Engineering, Dartmouth College, Hanover, New Hampshire, USA; c Department of Pathology, Johns Hopkins School of Medicine, Baltimore, Maryland, USA; d W. Harry Feinstone Department of Molecular Microbiology and Immunology, Johns Hopkins Bloomberg School of Public Health, Baltimore, Maryland, USA; e Department of Pediatrics, Geisel School of Medicine at Dartmouth, Dartmouth-Hitchcock Medical Center, Lebanon, New Hampshire, USA; f Department of Medicine, Division of Infectious Diseases, Johns Hopkins School of Medicine, Baltimore, Maryland, USA; g Division of Intramural Research, National Institute of Allergy and Infectious Diseases, National Institutes of Health, Bethesda, Maryland, USA; Washington University School of Medicine

**Keywords:** convalescent plasma, SARS-CoV-2, COVID-19, neutralization, functional antibody response, ADCC, phagocytosis, IgA, IgG, antibody

## Abstract

Convalescent plasma is a promising therapy for coronavirus disease 2019 (COVID-19), but the antibody characteristics that contribute to efficacy remain poorly understood. This study analyzed plasma samples from 126 eligible convalescent blood donors in addition to 15 naive individuals, as well as an additional 20 convalescent individuals as a validation cohort. Multiplexed Fc Array binding assays and functional antibody response assays were utilized to evaluate severe acute respiratory syndrome coronavirus 2 (SARS-CoV-2) antibody composition and activity. Donor convalescent plasma samples contained a range of antibody cell- and complement-mediated effector functions, indicating the diverse antiviral activity of humoral responses observed among recovered individuals. In addition to viral neutralization, convalescent plasma samples contained antibodies capable of mediating such Fc-dependent functions as complement activation, phagocytosis, and antibody-dependent cellular cytotoxicity against SARS-CoV-2. Plasma samples from a fraction of eligible donors exhibited high activity across all activities evaluated. These polyfunctional plasma samples could be identified with high accuracy with even single Fc Array features, whose correlation with polyfunctional activity was confirmed in the validation cohort. Collectively, these results expand understanding of the diversity of antibody-mediated antiviral functions associated with convalescent plasma, and the polyfunctional antiviral functions suggest that it could retain activity even when its neutralizing capacity is reduced by mutations in variant SARS-CoV-2.

## Abstract (precis)

Convalescent plasma has been deployed globally as a treatment for COVID-19, but efficacy has been mixed. Better understanding of the antibody characteristics that may contribute to its antiviral effects is important for this intervention as well as offer insights into correlates of vaccine-mediated protection.

## INTRODUCTION

Since its emergence in 2019, severe acute respiratory syndrome coronavirus 2 (SARS-CoV-2) has spread rapidly and infected over 115 million individuals worldwide. As the medical community has mobilized to identify effective therapies to combat the virus, treatment with convalescent plasma derived from individuals who have recovered from coronavirus disease 2019 (COVID-19) has emerged as a potential therapeutic intervention (1, 2). Preliminary evidence suggests that patients treated early with convalescent plasma show improved survival and reduced viral load, but efficacy data have been mixed (1, 3–10). While the Expanded Access Program demonstrated the efficacy of convalescent plasma in a dose-response effect of neutralizing titers, units with low titers were also efficacious, suggesting that there are other contributing activities that have not been measured (11). Antibody responses resulting from infection are highly variable in magnitude and character (12–14). Thus, a better understanding of the breadth and spectrum of antiviral activities of the humoral immune response is critical to understanding convalescent plasma as well as offering insights into correlates of vaccine-mediated protection.

Antibody responses to SARS-CoV-2 have exhibited wide variation, not only in titer but also in the quality of the antibody response including neutralization potential (14–18). Antibodies directed against the receptor-binding domain of the fusogenic spike (S) protein can block the interaction of the spike with the angiotensin-converting enzyme 2 (ACE2) receptor of airway epithelial cells. Such neutralizing antibodies have demonstrated the ability to inhibit infection *in vitro* (19, 20) and *in vivo* (21–23). Accordingly, neutralizing responses have been a key target in development of vaccines to prevent SARS-CoV-2 and monoclonal antibodies to treat COVID-19 disease (18). Recent data suggest that the frequency of neutralizing antibodies (nAbs) within the total humoral response could be quite low (24, 25) and that many antibodies are directed toward nonneutralizing epitopes within more conserved regions of the S protein (26–28). In the absence of sufficient levels of direct antiviral activity via antibody (Ab)-mediated blocking, the burden for humoral protection falls to the extraneutralizing effector functions, which are initiated by the relatively constant domain (Fc) of virus-specific antibodies and executed by innate immune cells and the complement cascade. By engaging soluble and cell surface-expressed Fc receptors, antibodies can trigger a variety of functions such as phagocytosis, cellular cytotoxicity, and complement deposition, which play an important role in clearing diverse viral infections (29, 30). In the context of SARS-CoV-1, antibody-mediated phagocytosis has been observed to play a critical role in clearing infection *in vivo*, even in the context of existing potent neutralization activity (31).

Further, these activities, which in principle can be mediated by a greater diversity of epitope specificities, may gain in importance in the context of emerging neutralization-resistant viral variants (32–34). Even in the context of potent neutralization activity, there is growing appreciation of the role that effector functions play *in vivo*. Multiple passive transfer experiments have shown that effector mechanisms contribute to the antiviral activity of monoclonal antibodies (35–38) and polyclonal antibodies raised in the context of vaccination (39, 40). This evidence extends beyond correlative observations (37, 39) to include mechanistic evidence of *in vivo* contributions via Fc sequence engineering to knock out or enhance these activities (36, 38), as well as on the basis of depletion of effector cells (38). Such findings indicate that understanding the ability of convalescent donor plasma to elicit these effector functions in diverse infected subjects may contribute to efficacy.

## RESULTS

### Biophysical characterization of SARS-CoV-2 convalescent plasma.

Convalescent plasma samples from 126 eligible donors from the Baltimore/Washington, DC area (Johns Hopkins Medical Institutions [JHMI] cohort) (14) and serum samples from 15 naive controls and 20 convalescent subjects from New Hampshire (Dartmouth-Hitchcock Medical Center [DHMC] cohort) (41) serving as a validation cohort were collected (see Table S1 in the supplemental material). Antibody responses were evaluated using an Fc Array assay that assesses both variable fragment (Fv) and Fc domain characteristics of antibodies (42). The assay was customized to assess responses across a panel of SARS-CoV-2 antigens. This panel consisted of the nucleocapsid (N) protein, stabilized trimeric spike protein (S-2P) (43), spike subdomains, including S1 and S2, the receptor-binding domain (RBD), and the fusion peptide. Influenza hemagglutinin (HA) and herpes simplex virus gE antigens served as controls. Characterization extended beyond antigen specificity to include antibody isotype, subclass, and propensity to bind Fc receptors (FcRs).

10.1128/mBio.00765-21.5 TABLE S1 Demographic information on convalescent plasma (JHMI) and convalescent plasma samples (DHMC). Download Table S1, PDF file, 0.02 MB. Copyright © 2021 Natarajan et al. 2021 Natarajan et al. https://creativecommons.org/licenses/by/4.0/ This content is distributed under the terms of the Creative Commons Attribution 4.0 International license.

**FIG 1:**
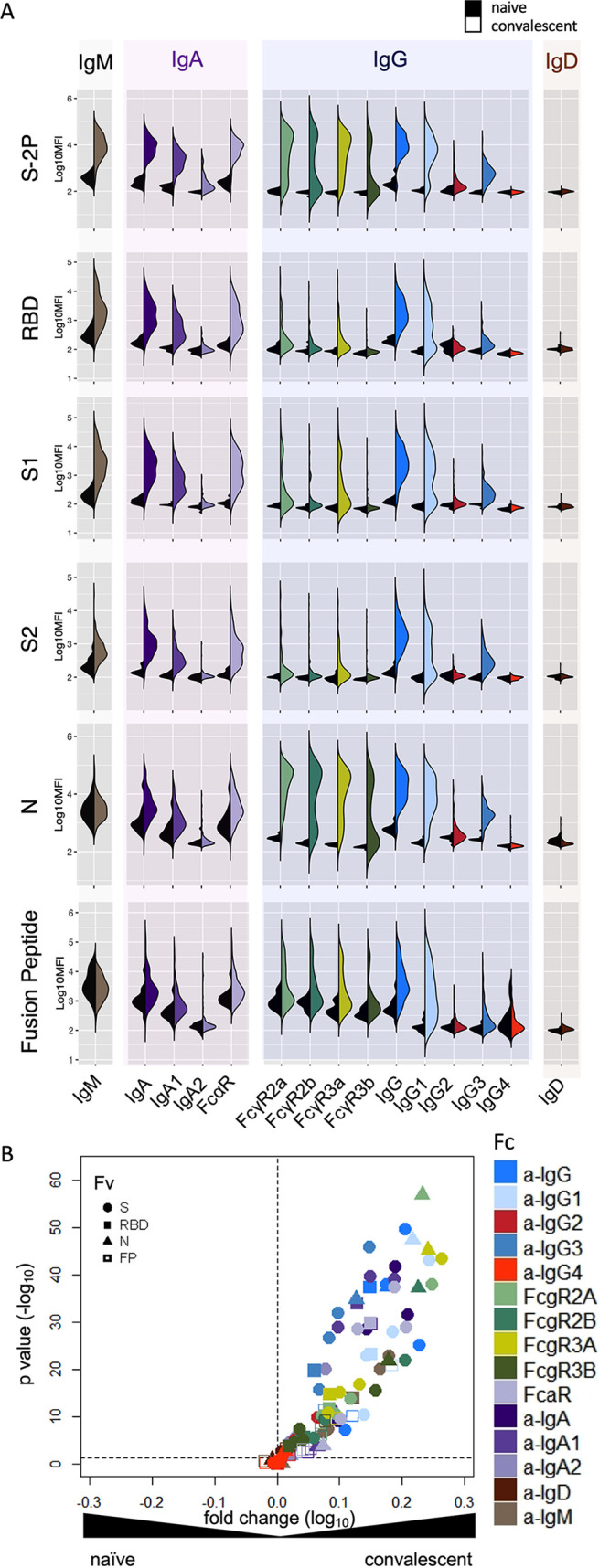
Antibody responses in convalescent plasma. (A) Fc Array characterization of antibodies to SARS CoV-2 antigens across antibody subclasses, isotypes, and binding to FcR in naive (serum; *n* = 15) and convalescent (plasma; *n* = 126) donors. (B) Volcano plot of fold change and significance of differences between convalescent and naive subject antibody response features specific for SARS-CoV-2.

Diverse SARS-CoV-2-specific immunoglobulin isotypes and subclasses, particularly IgG1 and IgG3, IgA, and IgM, were elevated in SARS-CoV-2 convalescent subjects across different epitope and antigen specificities (Fig. 1A; see also Fig. S1 to S3 in the supplemental material). Robust responses to stabilized spike (S-2P) and N were apparent, and lower magnitudes of responses were detected to functionally relevant RBD and fusion peptide domains. IgA responses were primarily driven by the IgA1 subclass, whereas IgG responses were dominated by IgG1 and IgG3. FcγR binding profiles, which capture the effects of other factors known to impact avid FcγR recognition, such as binding to multiple epitopes, distinct spatial recognition profiles, and differing antibody glycosylation, were distinct from measurements of the overall SARS-CoV-2-specific IgG response (titer), as well as compared to these innate immune activating IgG subclasses.

10.1128/mBio.00765-21.1 FIG S1 Plasma (JHMI) and serum (DHMC) IgG isotype and subclass responses across SARS-CoV-2 and control antigens. Samples from convalescent (+) donors are indicated in black and green, and samples from SARS-CoV-2 naive (−) subjects are indicated in red. Download FIG S1, PDF file, 0.3 MB. Copyright © 2021 Natarajan et al. 2021 Natarajan et al. https://creativecommons.org/licenses/by/4.0/ This content is distributed under the terms of the Creative Commons Attribution 4.0 International license.

### Relationship between antibody characteristics and clinical characteristics.

**FIG 2:**
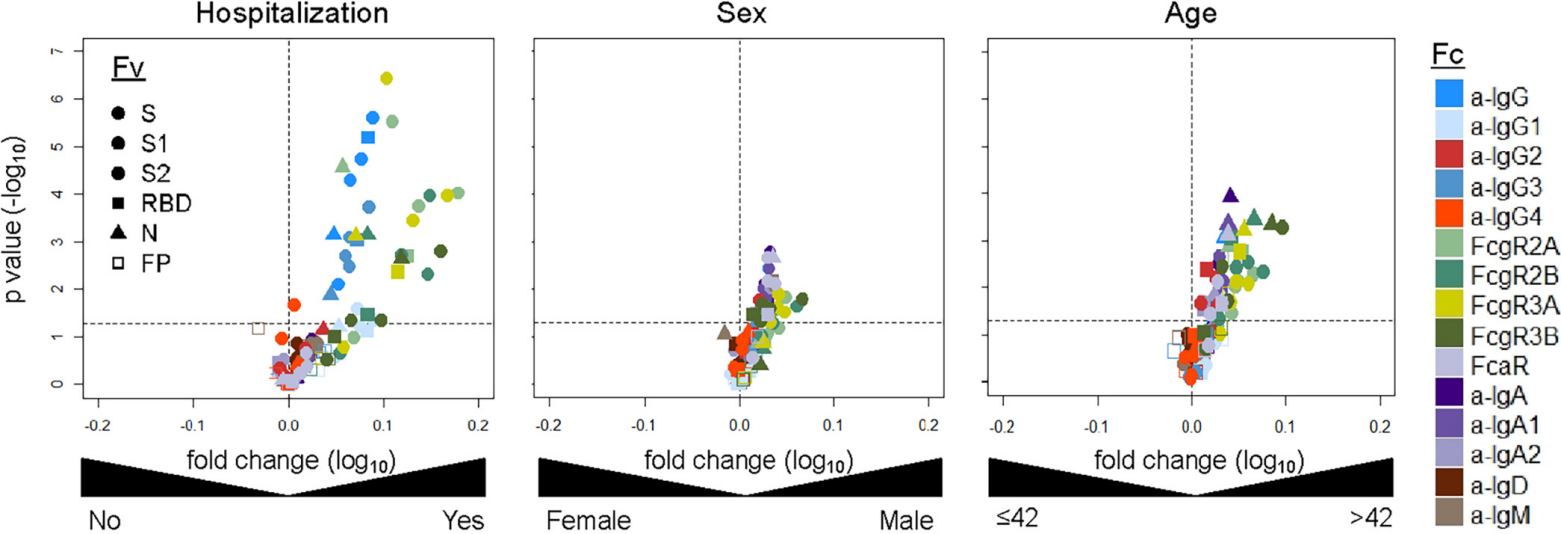
Antibody features associated with clinical status. Volcano plots of significance and fold change of response medians for antibody responses to SARS-CoV-2 antigens according to hospitalization status (left), sex (center), and age above or below the cohort median (right). Symbol shapes indicate Fv specificity, and color indicates Fc characteristic.

Differences in the antibody response toward SARS-CoV-2 and endemic CoV associated with sex, age, and hospitalization status were evaluated (Fig. 2). A chi-squared test was performed to examine whether there was an association between disease severity and gender. It was determined that there was no statistically significant link between these two traits (*P* = 0.7355). Moreover, a univariate logistic regression analysis was employed to assess the association between age and either gender of hospitalization status (sex: age, *P* = 0.711; hospitalization: age, *P* = 0.593). Therefore, the potential confounding effect on the association between biophysical measurements and each clinical trait were eliminated. SARS-CoV-2 IgG, IgA, and FcγR-binding antibodies were significantly elevated in older and male subjects, characteristics which are considered risk factors for more severe disease. Elevated SARS-CoV-2-specific IgG and FcγR-binding antibody features were also observed in hospitalized subjects, consistent with prior studies (14, 41, 44), and it is possible that IgG responses may drive disease enhancement (45–47). However, despite being associated with both age and sex risk factors, elevated IgA features were not observed in hospitalized subjects, consistent with the possibility that IgA responses may contribute to milder infection (41).

### Antibody effector functions.

**FIG 3:**
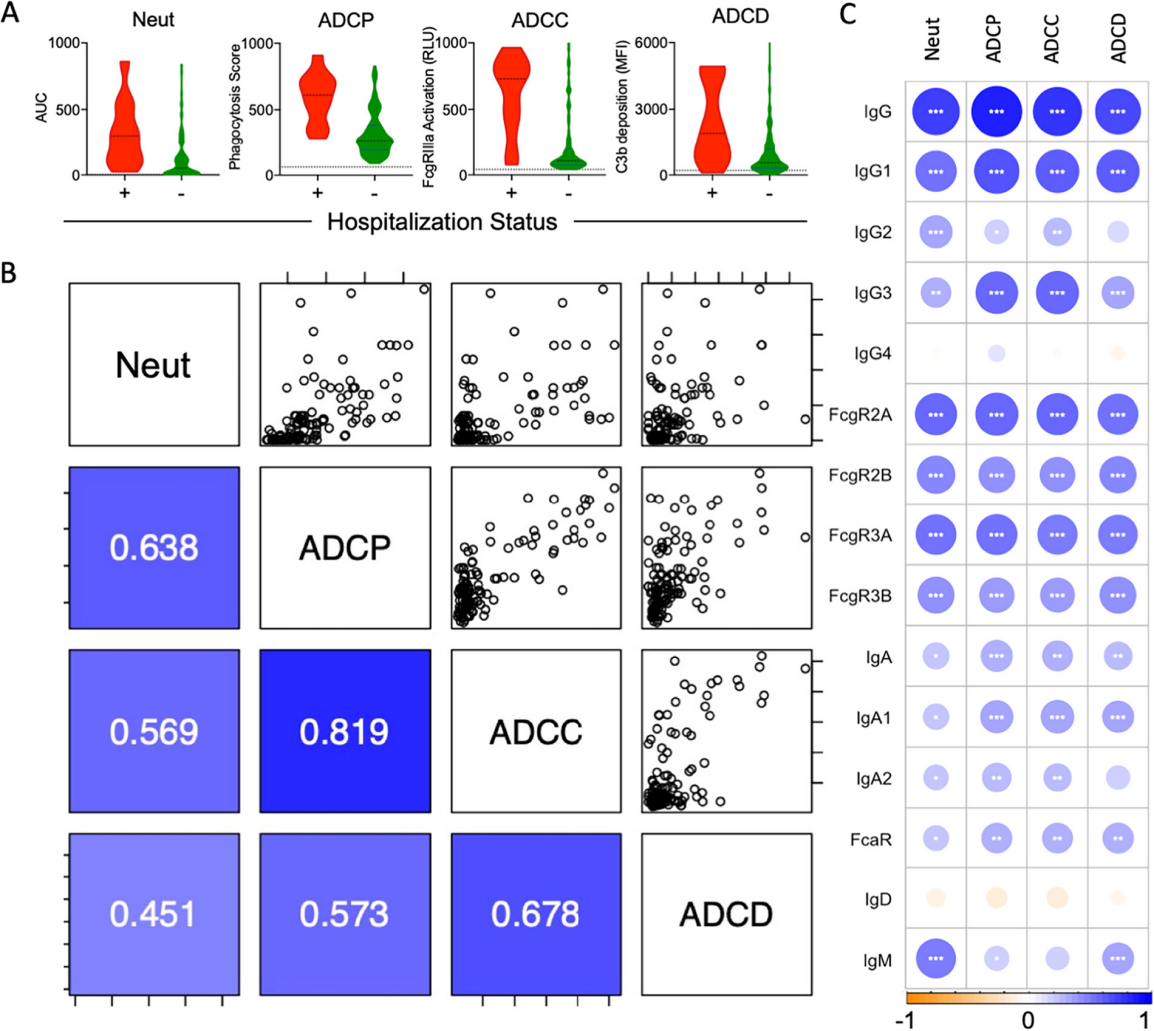
Functional characterization of plasma antibodies. (A) Neutralization (Neut), antibody-dependent cell-mediated phagocytosis (ADCP), FcγRIIIa activation (ADCC), and antibody-dependent complement deposition (ADCD) activity of convalescent plasma donors that were hospitalized (+) (*n* = 12) or not hospitalized (−) (*n* = 114). The dotted line indicates mean activity observed among naive donor samples. (B) Correlations between RBD-specific Ab features to functions in plasma, colored and labeled by Pearson correlation coefficient (*R_P_*) in the lower-left quadrant (*n* = 126). (C) Correlations (*R_P_*) between RBD-specific Fc Array features and neutralization and effector functions. Significance of Pearson correlations, corrected for multiple comparisons using the Benjamini-Hochberg method (*, *P* < 0.05; **, *P* < 0.01; ***, *P* < 0.001), are provided along with circles that are colored and sized according to their Pearson correlation coefficients (*R_P_*).

To explore the biological functions of antibodies in convalescent serum donors, both neutralizing and extraneutralizing activities were evaluated (Fig. 3A). Consistent with the overall SARS-CoV-2 antibody response magnitude, neutralization activity against live virus was higher among donors who were hospitalized than those who were not hospitalized. While antibody-dependent cell-mediated phagocytosis (ADCP), FcγRIIIa activation as a surrogate for antibody-dependent cellular cytotoxicity (ADCC), and antibody-dependent complement deposition (ADCD) elicited by RBD-specific antibodies were likewise elevated among donors who had been hospitalized, correlative relationships between functions showed distinctions among these antiviral activities (Fig. 3B). ADCP, which has previously been associated with clearance of SARS-CoV-1 in a mouse model (31), and FcγRIIIa activation (ADCC) were highly correlated with each other (Pearson correlation coefficient [*R_P_*] = 0.82), and moderately correlated with neutralization (*R_P_* = 0.64 and 0.57, respectively). Complement activation, which has been associated with increased inflammation and disease pathology in a mouse model of SARS-CoV-1 (48) and which may also contribute to COVID-19 disease (49, 50), was less well correlated with other activities.

### Antibody features associated with antiviral functions.

Because a number of the effector functions were tested specifically against the RBD antigen, we measured the degree and direction of correlation between RBD-specific Ab biophysical features and other Ab functions (Fig. 3C). ADCP and FcγRIIIa activation (ADCC) were most strongly correlated with FcγR-binding antibodies, and IgG1 and IgG3, which strongly ligate FcγR. Among FcR, correlations with activating FcγRIIa and FcγRIIIa were most strongly correlated, consistent with their known mechanistic relevance to ADCP and FcγRIIIa activation (ADCC).

As previously observed (41), IgM also positively correlated with neutralization activity. Relationships between serum IgA responses and antibody functions were considerably weaker than those with IgG responses. Correlative relationships with ADCD tended to be weaker, consistent with the strong dependence of this function on spatial aspects of avid antibody binding and immune complex formation that are better captured by detection with the C1q, an initiator of the complement cascade that was not evaluated in this study.

### Distinctions between subjects defined by humoral response profiles.

**FIG 4:**
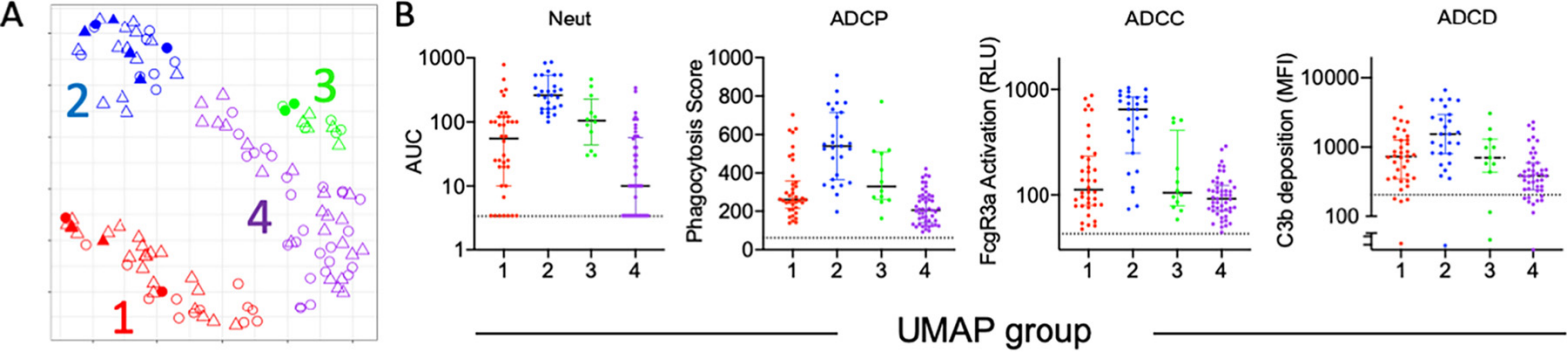
Identification of polyfunctional plasma samples. (A) UMAP analysis of subjects (*n* = 126) based on Fc Array antibody biophysical profiles. The position in variable space indicates similarity or distinctions in antibody response. Symbols and color indicate subject sex, hospitalization status, and cluster. (B) Boxplots depicting the level of antibody functions observed among subjects in each UMAP group. Polyfunctional plasma donors are observed among UMAP group 2. Dotted lines indicate mean activity observed among naive donor samples.

To define similarities and differences among donors more globally, dimensionality reduction was performed on biophysical features using Uniform Manifold Approximation and Projection (UMAP) (51). Subjects were distributed across the antibody biophysical profile landscape into a set of four distinct clusters (Fig. 4A). Though hospitalized subjects were observed in multiple clusters, they were most prevalent in cluster 2 and adjacent regions of clusters 1 and 3. To understand aspects of the humoral response that distinguished each cluster, univariate testing was performed to determine and depict which Fc Array features were distinct for individual clusters (Fig. S4). Relative responses for these features among convalescent donors in each group reflect differences in the magnitude of the response, with cluster 1 having lower humoral responses to SARS-CoV-2 antigens in general, clusters 2 and 3 exhibiting intermediate responses, and cluster 4 typically showing globally elevated antibody responses. Clusters 2 and 3, which both presented with intermediate response magnitudes, were distinguished by relative differences in IgG1 and IgA responses (Fig. S4).

10.1128/mBio.00765-21.2 FIG S2 Plasma (JHMI) and serum (DHMC) IgA, IgD, and IgM isotype and subclass responses across SARS-CoV-2 and control antigens. Samples from convalescent (+) donors are indicated in black and green, and samples from SARS-CoV-2 naive (−) subjects are indicated in red. Download FIG S2, PDF file, 0.4 MB. Copyright © 2021 Natarajan et al. 2021 Natarajan et al. https://creativecommons.org/licenses/by/4.0/ This content is distributed under the terms of the Creative Commons Attribution 4.0 International license.

10.1128/mBio.00765-21.3 FIG S3 Plasma (JHMI) and serum (DHMC) FcγR binding responses across antibodies specific to SARS-CoV-2 and control antigens. Samples from convalescent (+) donors are indicated in black and green, and samples from SARS-CoV-2 naive (−) subjects are indicated in red. Download FIG S3, PDF file, 0.3 MB. Copyright © 2021 Natarajan et al. 2021 Natarajan et al. https://creativecommons.org/licenses/by/4.0/ This content is distributed under the terms of the Creative Commons Attribution 4.0 International license.

10.1128/mBio.00765-21.4 FIG S4 Heatmap of distinct features by UMAP group. Heatmap of filtered (*P* < 0.05 among groups) and hierarchically clustered Fc Array features in serum (left) and nasal wash (right) across subjects with differing infection or disease status. Each row represents an individual donor. Disease severity is shown on the left annotation bar. Each column represents an Fc Array measurement, with antigen specificity (Fv) and Fc characteristics (Fc) are indicated in top color bars. Responses are centered and scaled per feature, and the scale range is truncated at ±3 SD. Relatively high responses are indicated in red, and low responses are indicated in blue. Download FIG S4, PDF file, 0.2 MB. Copyright © 2021 Natarajan et al. 2021 Natarajan et al. https://creativecommons.org/licenses/by/4.0/ This content is distributed under the terms of the Creative Commons Attribution 4.0 International license.

### Identification of polyfunctional plasma samples.

**FIG 5:**
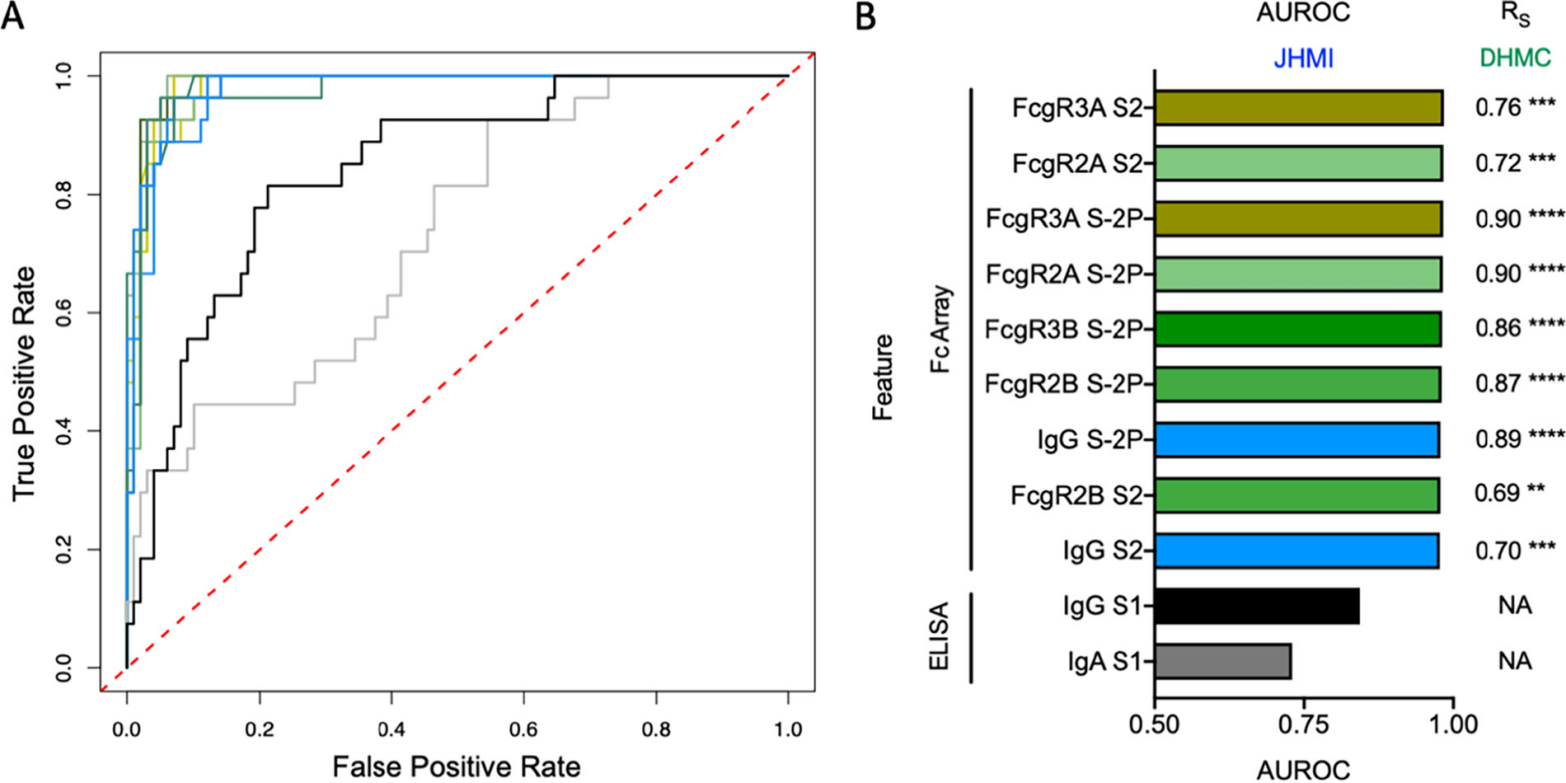
Identification and validation of antibody response features that identify polyfunctional plasma. (A) Receiver operator characteristic curve for the ability of selected features to discriminate between polyfunctional plasma samples (UMAP group 2) and nonpolyfunctional plasma samples (UMAP groups 1, 3, and 4). Features in panel A are colored according to assay and detection reagent type, as in panel B. (B) Area under the ROC (AUROC) curve for discrimination of polyfunctional plasma among JHMI cohort (*n* = 126) for the top Fc Array features and clinical ELISAs. Spearman correlation coefficients (*R_S_*) between these features and polyfunctionality score in the DHMC validation cohort (*n* = 20) are given at right. Significance of Spearman correlations, corrected for multiple comparisons using the Benjamini-Hochberg method, are provided (**, *P* < 0.01; ***, *P* < 0.001; ****, *P* < 0.0001). ELISA data were not available (NA) for the DHMC cohort.

Based on the potential clinical value of polyfunctional plasma, the neutralization and effector functions of each subject according to their UMAP cluster was defined (Fig. 4B). Subjects in cluster 2 exhibited elevated activity across diverse functions, identifying this cluster of donors as possessing polyfunctional plasma. Because screening plasma for multiple individual functional activities is impractical, we sought to identify the ability of individual Fc Array measurements and classical clinical enzyme-linked immunosorbent assay (ELISA) tests to identify polyfunctional plasma samples. To determine which features were able to distinguish polyfunctional (cluster 2) from nonpolyfunctional (clusters 1, 3, and 4), receiver operator characteristic (ROC) curves were generated for each Fc Array feature and available clinical ELISA. This analysis identified SARS-CoV-2 S-2P- and S2-specific IgG and FcγR-binding antibodies as exhibiting good discriminatory capacity (Fig. 5A). Area under the ROC curve for the top nine Fc Array features was superior to that of S1-based clinical ELISAs (Fig. 5B).

To validate this observation, the strength and significance of correlations between these features and a polyfunctionality score for the serum samples from the validation cohort was evaluated (Fig. 5B). Strong and significant correlations were observed for both FcγR-binding antibodies specific to stabilized spike (S-2P) and the S2 domain, as well as the magnitude of total IgG responses to these proteins. Together, discovery and validation cohorts support the ability of individual high-throughput multiplex assay measurements to predict the polyfunctionality of donor plasma samples. Identification of highly and broadly antiviral donor plasma samples has the potential to improve treatment outcomes or prevent reinfection.

## DISCUSSION

Convalescent plasma is one of the leading treatments of hospitalized patients for COVID-19. Following transfusion of more than 100,000 individuals in the United States with convalescent plasma, the FDA issued an expanded use authorization. In general, convalescent plasma appears to be most effective when high-titer units are provided early, but efficacy data have been mixed (5, 10). Thus, it is important to establish the specific, measurable qualities of convalescent plasma that may help us to understand the mechanism of action and resistance of convalescent donors to reinfection.

SARS-CoV-2-specific antibodies can elicit diverse antiviral functions beyond neutralization. These less well characterized functions were measured and related to biophysical antibody profiles. Effector functions were most strongly correlated with FcγR-binding antibodies, IgG1, and IgG3. Beyond IgG, neutralization was also correlated with IgM responses, which may suggest the development of novel responses, as opposed to reactivation of responses to endemic CoV. SARS-CoV-2- specific IgM has also attracted interest because of its association with lower risk of death from COVID-19 (12), and recent evidence of direct neutralization activity (52, 53). Evidence in support of the relevance of nonneutralizing mechanisms of antibody-mediated protection against SARS-CoV-2 is accruing (35–40), and there is evidence that both ADCC and phagocytosis can contribute antiviral effects against other coronaviruses (54–56). Thus, activities measured by these and other *in vitro* assays have demonstrated the ability to play important roles in antibody-mediated defense against SARS-CoV-2, and as observed in a number of studies in other infectious disease settings (57–62).

The antibody responses measured in convalescent subjects in this study were highly diverse, both in the SARS-CoV-2 antigens recognized and the magnitude of the responses; the latter observation is largely characteristic of the humoral responses measured to date (12, 13). Some of these differences are likely associated with the different manifestations of disease, but others may relate to the cross-sectional nature of the cohort and factors such as differences in time since infection. Other limitations of this study range from cohort composition to the experimental and analytical approaches employed. Individuals in the naive control cohort were generally younger and from a different geographic location. Recombinant antigen and lab-adapted cell lines were employed for several of the functional assays, and the substitution of surrogate measurements such as FcγRIIIa activation was made in place of infected cell death. Thus, while the value of such assays has been established in a diversity of other infectious disease settings (57–62), as well as in SARS-CoV-2 in animal models (35–40), *in vitro* function may differ substantially from the *in vivo* processes these assays are meant to mimic. Though performed on dilute plasma, these assay results could also include effects of plasma components other than antibodies, such as cytokines and complement factors, which cannot be disambiguated from the data collected, and may or may not be relevant to the effects of convalescent plasma therapy (63). Additionally, the convalescent and naive subjects enrolled in the DHMC cohort provided serum samples, whereas the convalescent subjects in the JHMI cohort contributed plasma samples, which could result in differences in antibody detection and functional activity. Nevertheless, the Fc Array feature that best identified polyfunctional convalescent-phase plasma samples was also able to accurately identify polyfunctional convalescent-phase serum samples.

In summary, this study establishes three Fc-dependent activities in convalescent plasma beyond viral neutralization that could have antiviral effects against SARS-CoV-2, namely, ADCC, phagocytosis, and complement activation. These activities could explain therapeutic effects of plasma samples with low neutralizing capacity (11). With this study we also provide a proof of principle for the correlation of diverse antiviral activities against SARS-CoV-2 using biophysical inputs more amenable to high-throughput measurement. The recent futility-based termination of the ACTIV-3 clinical trial of neutralizing antibody bamlanivimab in hospitalized subjects points to potential challenges to therapy based on individual monoclonal antibodies. Taken together, it is possible that optimally effective antibody therapies for SARS-CoV-2 may require a polyclonal antibody response, which can control virus through binding to multiple epitopes and eliciting a range of effector functions. This work begins to define the specificities and Fc domain characteristics of antibodies associated with potent neutralization as well as effector function and to distill a means to identify polyfunctional plasma samples from a single high-throughput multiplex assay readout.

Further insights into the antibody response to COVID-19 among convalescent plasma donors may be of special interest due to the emergence of novel variants of SARS-CoV-2. Whereas convalescent plasma has shown compromised neutralization capability against novel variants (33, 34), other effector functions that are typically effectively driven by a broad diversity of epitope specificities may prove less sensitive to neutralization escape mutants or other forms of antigenic drift. However, therapeutically desirable plasma antibody functions have yet to be determined in humans. A strong evidence base exists for the role of antibodies in protection based on animal models and in the setting of human immune responses against other CoV (64–66) and SARS-CoV-2 (67, 68). This evidence now includes observations of both beneficial as well as detrimental potential consequences of diverse effector functions (35–40, 69, 70), suggesting that continued analysis of the associations between passively transferred plasma characteristics and patient outcomes will be key to identifying the recipients who are most likely to benefit and the donors most likely to provide that benefit in the context of the COVID-19 pandemic.

## MATERIALS AND METHODS

### Human subjects.

The principal cohort of the study has been previously described (14). Briefly, it comprised 126 adult subjects (mean age, 43 years; range, 19 to 78 years) previously diagnosed with SARS-CoV-2 infection by PCR-positive (PCR+) nasal swab who met the standard eligibility criteria for blood donation and were collected in the Baltimore, MD and Washington, DC area (Johns Hopkins Medical Institutions [JHMI] cohort). The cohort was composed of 68 males (54.0%) and 58 females (46.0%). Eleven cases (8.7%) were severe enough to require hospitalization (mean duration of stay, 4 days; range, 1 to 8 days). The validation cohort comprised 20 SARS-CoV-2 convalescent individuals from the Hanover, New Hampshire area (Dartmouth Hitchcock Medical Center [DHMC] cohort) (mean age, 40 years; range, 18 to 77 years) and comprised 10 males and 10 females among which 4 subjects (20%) were hospitalized. Infection with SARS-CoV-2 was confirmed in all convalescent subjects by real-time reverse transcriptase polymerase chain reaction of a nasopharyngeal swab. Plasma (JHMI) or serum (DHMC) was collected from each donor approximately 1 month after symptom onset or first positive PCR test in the case of mild or asymptomatic disease (see Table S1 in the supplemental material).

Human subject research was approved by both the Johns Hopkins University School of Medicine’s Institutional Review Board and the Dartmouth-Hitchcock Medical Center Committee for the Protection of Human Subjects. All participants provided informed written consent.

### Antigen and Fc receptor expression and purification.

Prefusion-stabilized, trimer-forming spike protomers (S-2P) of SARS-CoV-2 and a fusion of the receptor-binding domain of SARS-CoV-2 N terminally to a monomeric human IgG4 Fc domain were transiently expressed in either Expi 293 or Freestyle 293-F cells and purified via affinity chromatography, all according to the manufacturers’ protocols, as previously described (41). Human FcγR were expressed and purified as described previously (71).

### Fc Array assay.

Coronavirus antigens—including S trimers, S subdomains (i.e., S1 and S2), and other viral proteins from SARS-CoV-2 (Table S2)—and the control antigens influenza HA and herpes simplex virus (HSV) gE proteins were covalently coupled to Luminex Magplex magnetic microspheres using two-step carbodiimide chemistry as previously described (70). Biotinylated SARS-CoV-2 fusion peptide was immobilized on neutravidin-coupled microspheres. Pooled polyclonal serum IgG (intravenous immunoglobulin [IVIG]), the SARS CoV-1-specific monoclonal Ab CR3022 that cross-reacts with SARS-CoV-2 S (72), and VRC01, an HIV-specific monoclonal Ab, were used as controls to define bead antigenicity profiles. Pilot experiments were used to determine the optimal dilution of plasma for titrations. Test concentrations for plasma ranged from 1:250 to 1:5,000 and were varied per detection reagent (Table S3). Isotypes and subclasses of antigen-specific Abs were detected using R-phycoerthrin (PE)-conjugated secondary Abs and by FcRs tetramers as previously described (73). A FlexMap three-dimensional (3D) array reader detected the beads and measured PE fluorescence in order to calculate the median fluorescence intensity (MFI).

10.1128/mBio.00765-21.6 TABLE S2 Antigen and Fc detection reagents. Download Table S2, PDF file, 0.02 MB. Copyright © 2021 Natarajan et al. 2021 Natarajan et al. https://creativecommons.org/licenses/by/4.0/ This content is distributed under the terms of the Creative Commons Attribution 4.0 International license.

10.1128/mBio.00765-21.7 TABLE S3 Sample dilutions in the Fc Array assay. Download Table S3, PDF file, 0.02 MB. Copyright © 2021 Natarajan et al. 2021 Natarajan et al. https://creativecommons.org/licenses/by/4.0/ This content is distributed under the terms of the Creative Commons Attribution 4.0 International license.

### Neutralization assays.

Plasma samples from SARS-CoV-2 convalescent donors were tested in microneutralization assays using SARS-CoV-2/WA-1/2020 virus (14, 74) obtained from BEI Resources. VeroE6-TMPRSS2 cells were used to propagate the virus and to determined infectious virus titers using a 50% tissue culture infectious dose (TCID_50_) assay as previously described for SARS-CoV (14, 74) using Institutional Biosafety Committee-approved protocols in biosafety level 3 containment. Twofold dilutions of plasma were incubated with 100 TCID_50_s for 1 h at room temperature in a volume of 100 μl. The virus-plasma solution was then added to one well of VeroE6-TMPRSS2 cells in a 96-well plate and incubated for 6 h before being replaced with medium. After incubation at 37°C for 2 days, the cells were fixed with 150 μl of 4% formaldehyde followed by staining with Naptho blue black (Sigma-Aldrich) and scoring for wells protected from infection. The assay was performed in hextuplicate, and the area under the curve was calculated from the neutralizing antibody curve. Neutralization of the serum samples was tested using a vesicular stomatitis virus (VSV)-SARS-CoV pseudovirus system as previously described (41, 75), and neutralization was expressed as 60% inhibitory concentration (IC_60_) values.

### Phagocytosis assay.

An assay of Ab-dependent phagocytosis by monocytes (ADCP) was performed essentially as described previously (76, 77). Briefly, 1-μm yellow-green fluorescent microspheres (Thermo catalog no. F8813) covalently conjugated with recombinant RBD were incubated for 3 h with dilute plasma specimens and the human monocytic THP-1 cell line (ATCC, TIB-202). After pelleting, washing, and fixing, phagocytic scores were calculated as the product of the percentage of cells that phagocytosed one or more fluorescent beads and the median fluorescent intensity of this population was measured by flow cytometry with a MACSQuant analyzer (Miltenyi Biotec). CR3022 and VRC01 were used as positive and negative controls, respectively. Antibody-independent phagocytosis was measured from wells containing cells and beads, but no antibody.

### FcγRIIIa activation reporter assay.

A Jurkat Lucia NFAT reporter cell line (Invivogen, jktl-nfat-cd16) was used to measure the ADCC potential, represented by the extent of FcγRIIIa activation, of each sample. Engagement of the cell surface receptor leads to the secretion of luciferase into the cell culture supernatant. The cells were cultured according to the manufacturer’s recommendations. One day prior to performing the assay, a high binding 96-well plate was coated with 1 μg/ml SARS-CoV-2 RBD and incubated at 4°C overnight. The plates were then washed with phosphate-buffered saline (PBS) plus 0.1% Tween 20 and blocked at room temperature for 1 h with PBS plus 2.5% bovine serum albumin (BSA). After washing, dilute plasma and 100,000 cells/well in growth medium lacking antibiotics (with a total volume of 200 μl) were cultured at 37°C for 24 h. The following day, 25 μl of supernatant was drawn from each well and transferred to an opaque, white 96-well plate and 75 μl of QuantiLuc substrate was added. Luminescence was read immediately on a SpectraMax Paradigm plate reader (Molecular Devices) using 1 s of integration time. The reported values are the means of three kinetic reads taken at 0, 2.5, and 5 min. Negative-control wells contained assay medium instead of antibody sample, while cell stimulation cocktail (Thermo catalog no. 00-4970-93) plus an additional 2 μg/ml ionomycin were used to induce expression of the luciferase transgene as a positive control.

### Complement deposition assay.

Antibody-dependent complement deposition (ADCD) was quantified essentially as previously described (78). In brief, plasma samples were heat inactivated at 56°C for 30 min prior to a 2-h incubation with multiplex assay microspheres at room temperature. After washing, each sample was incubated for 1 h at room temperature with human complement serum (Sigma catalog no. S1764) at a concentration of 1:50. Samples were washed, sonicated, and incubated for 1 h at room temperature with murine anti-C3b (Cedarlane catalog no. CL7636AP) followed by anti-mouse IgG1-PE secondary Ab (Southern Biotech catalog no. 1070-09) at room temperature for 30 min. After a final wash and sonication, samples were resuspended in Luminex Sheath Fluid and complement deposition in the form of the median fluorescent intensity of the PE measured on a MAGPIX (Luminex Corp.) instrument. Wells lacking Ab and but still containing heat-inactivated human complement serum served as negative controls.

### Data analysis and visualization.

Basic analysis and visualization were performed using GraphPad Prism. Heatmaps, correlation plots, and boxplots were generated in R version 3.6.1 (supported by R packages pheatmap [79], corrplot [80], and ggplot2 [81]). Hierarchical clustering was used to cluster and visualize data using the Manhattan and Euclidean metrics. Fc Array features were filtered by elimination of features for which the samples exhibited signal within 10 standard deviations (SD) of the technical blank. Fc Array features were log transformed and then scaled and centered by their standard deviation from the mean (z-score). A Student’s two-tailed *t* test with Welch’s correction with a cutoff of *P* = 0.05 was used to define features different between groups. Pearson correlation coefficients were calculated for the correlation matrices. Spearman correlation coefficients were used to relate antibody features to polyfunctionality scores. The Benjamini-Hochberg method was used to adjust determinations of statistical confidence due to multiple hypothesis testing.

UMAP was employed in the R package “umap” version 0.2.6.0 (82) to enable dimensionality reduction of the JHMI Fc Array data set. Upon log transformation, default UMAP parameters were used with the following exceptions: random_state = 45, min_dist = 1E−9, knn_repeats: −1, set_op_mix_ratio = 1. DBSCAN (83) was employed in the R package “dbscan” version 01.1-5 to identify clusters within the UMAP reduced dimensions, using settings of eps = 0.65 and MinPts = 5 to define the clusters. To identify features associated with each cluster, individual clusters were compared to the other three clusters using a Student’s two-tailed *t* test with Welch’s and Bonferroni’s corrections and a cutoff of *P* = 0.05.

A receiver operating characteristic (ROC) curve was applied to evaluate the performance of biophysical features in discriminating polyfunctional plasma donors from nonpolyfunctional plasma donors. The binary labels were assigned among the subjects in cluster 2 (*n* = 27) versus the subjects in the rest of the clusters (*n* = 99). All biophysical features in the JHMI cohort were ranked by the area under the receiver operating characteristic (AUROC). R packages “ROCR” (84) and “pROC” (85) were employed for ROC curve generation and AUROC calculation.

A polyfunctionality score was defined for each validation (DHMC) cohort serum sample as follows. Minimum activities were assigned a value of zero, and maximum activity was assigned a value of one. Relative activity for each function was defined as (observed − minimum)/(maximum − minimum), and polyfunctionality was defined as the sum of these relative activity scores across functional assays.

### Availability of data.

Cohort characteristic, Fc Array, and functional assay data are available at https://github.com/AckermanLab/Natarajan_et_al_COVID_2021.
