# Supplementary material for: Markers of Polyfunctional SARS-CoV-2 Antibodies in Convalescent Plasma
Source: mBio. 2021 Apr 20;12(2):e00765-21. doi: 10.1128/mBio.00765-21 (PMC8092262; doi:10.1128/mBio.00765-21)
Supplement: TABLE S1 [file mBio.00765-21-st001.pdf]

Supplemental Table 1: Demographic information on convalescent plasma (JHMI) and convalescent plasma samples (DHMC).

**Characteristic**	**JHMI Convalescent** n=126	**DHMC Convalescent** n=20	**DHMC Naive** n=15
Median age (IQR), years	42 (29-53)	54 (45-62)	34 (28-52)
Sex			
Female	58 (46%)	10 (50%)	8 (53.3%)
Male	68 (54%)	10 (50%)	7 (46.7%)
Hospitalized (severity)			
No	114 (90.5%)	16 (80%)	NA
Yes	12 (9.5%)	4 (20%)	NA
Median days since PCR+ or symptom onset (IQR)	43 (38-48)	38 (33-45)	NA
