# Supplementary material for: Markers of Polyfunctional SARS-CoV-2 Antibodies in Convalescent Plasma
Source: mBio. 2021 Apr 20;12(2):e00765-21. doi: 10.1128/mBio.00765-21 (PMC8092262; doi:10.1128/mBio.00765-21)
Supplement: FIG S3 [file mBio.00765-21-sf003.pdf]

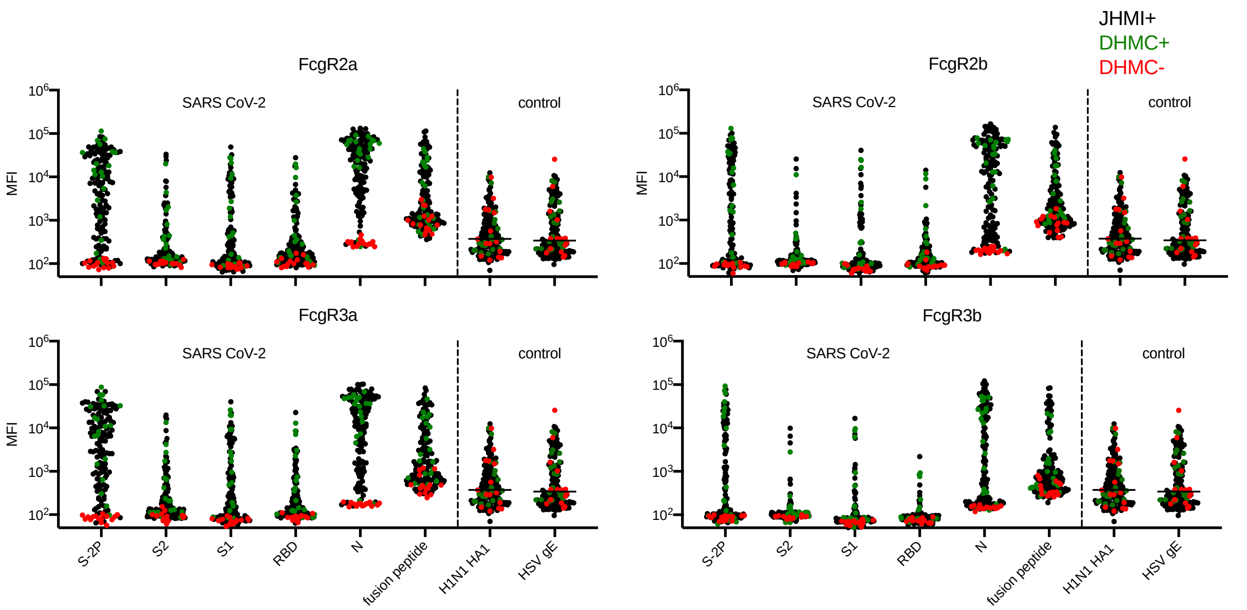

Supplemental Figure 3. Plasma (JHMI) and serum (DHMC) FcγR binding responses across antibodies specific to SARS-CoV-2 and control antigens. Samples from convalescent (+) donors are indicated in black and green, and those from SARS-CoV-2 naïve (-) subjects in red.
