# Supplementary material for: Markers of Polyfunctional SARS-CoV-2 Antibodies in Convalescent Plasma
Source: mBio. 2021 Apr 20;12(2):e00765-21. doi: 10.1128/mBio.00765-21 (PMC8092262; doi:10.1128/mBio.00765-21)
Supplement: FIG S4 [file mBio.00765-21-sf004.pdf]

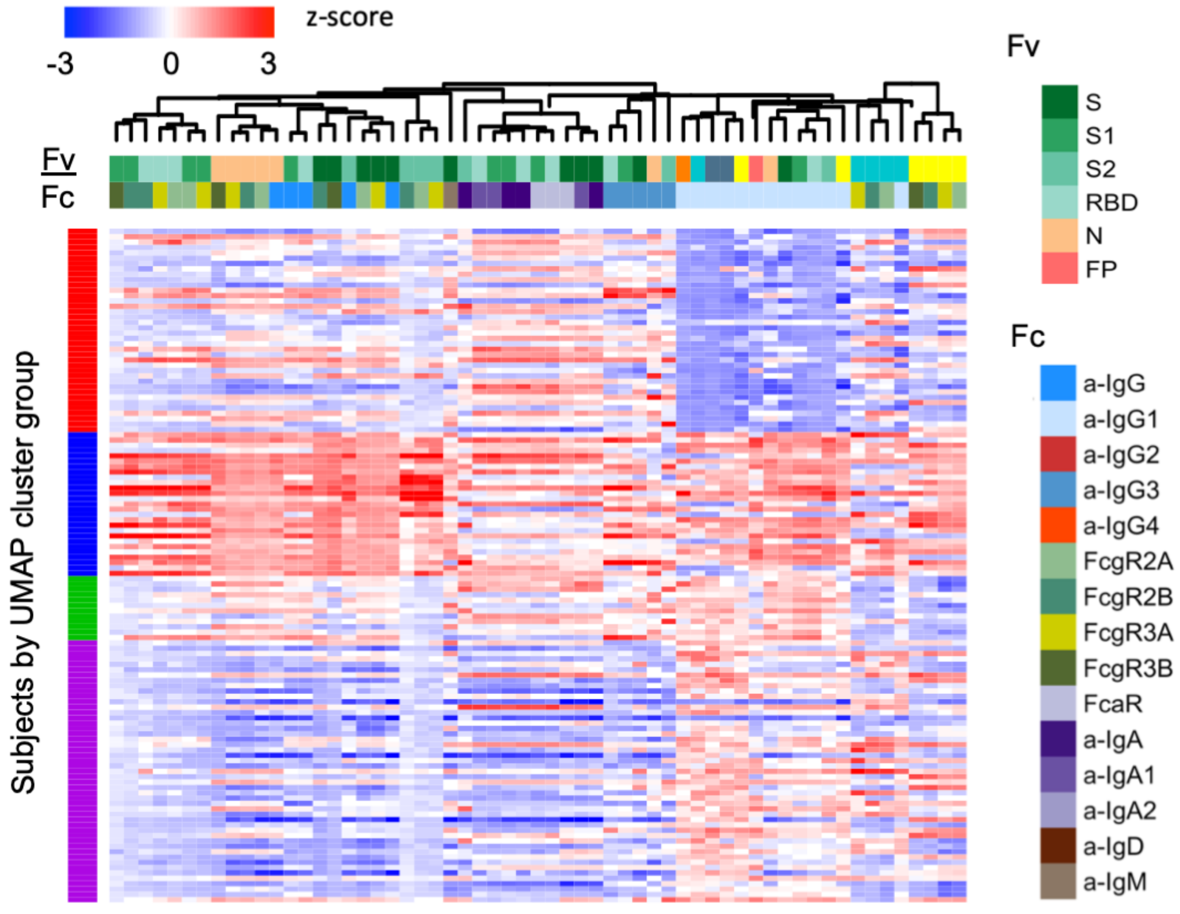

Supplemental Figure 4. Heatmap of distinct features by UMAP group. Heatmap of filtered ($p < 0.05$ among groups) and hierarchically-clustered Fc array features in serum (left) and nasal wash (right) across subjects with varying infection or disease status. Each row represents an individual donor. Disease severity is shown on the left annotation bar. Each column represents an Fc Array measurement, with antigen specificity (Fv) and Fc characteristics (Fc) are indicated in top color bars. Responses are centered and scaled per feature and the scale range truncated at +/-3 SD. Relatively high responses are indicated in red, and low responses in blue.
