# Supplementary material for: Markers of Polyfunctional SARS-CoV-2 Antibodies in Convalescent Plasma
Source: mBio. 2021 Apr 20;12(2):e00765-21. doi: 10.1128/mBio.00765-21 (PMC8092262; doi:10.1128/mBio.00765-21)
Supplement: TABLE S2 [file mBio.00765-21-st002.pdf]

Supplemental Table 2: Antigen and Fc Detection Reagents

Antigen	Source	Fc Detection	Source
H1N1 HA1	Immune Technology IT-003-00110p	a- IgG	Southern Biotech 1030-09
HSV gE	Immune Technology IT-005-005p	a-IgG1	Southern Biotech 9054-09
SARS CoV-2 N	Immune Technology IT-002-033Ep	a-IgG2	Southern Biotech 9070-09
SARS CoV-2 fusion peptide	New England Peptide	a-IgG3	Southern Biotech 9210-09
SARS CoV-2 S1 (for Fc Array)	ACROBiosystems S1N-C52H3-100ug	a-IgG4	Southern Biotech 9200-09
SARS CoV-2 RBD	BEI Resources NR-52366	a-IgA	Southern Biotech 2050-09
SARS CoV-2 S2	Immune Technology IT-002-034p	a-IgA1	Southern Biotech 9130-09
SARS CoV-2 S-2P	Expressed in Expi 293	a-IgA2	Southern Biotech 9140-09
		a-IgM	Southern Biotech 9020-09
		a-IgD	Southern Biotech 9030-09
		FcαR	Duke Protein Production Facility
		FcγR2a	Boesch, et. al, 2014[1]
		FcγR2b	Boesch, et. al, 2014[1]
		FcγR3a	Boesch, et. al, 2014[1]
		FcγR3b	Boesch, et. al, 2014[1]

[1]Boesch AW, Brown EP, Cheng HD, Ofori MO, Normandin E, Nigrovic PA, et al. Highly parallel characterization of IgG Fc binding interactions. MAbs. 2014;6(4):915-27.
