# Supplementary material for: Markers of Polyfunctional SARS-CoV-2 Antibodies in Convalescent Plasma
Source: mBio. 2021 Apr 20;12(2):e00765-21. doi: 10.1128/mBio.00765-21 (PMC8092262; doi:10.1128/mBio.00765-21)
Supplement: TABLE S3 [file mBio.00765-21-st003.pdf]

Supplemental Table 3. Sample Dilutions in the Fc Array Assay

**Sample Type**	**Dilution**	**Detection Reagent**
Serum and Plasma	1:5000	anti-human IgG, FcγR2A, FcγR2B, FcγR3A, FcγR3B
Serum and Plasma	1:1000	anti-human IgG1
Serum and Plasma	1:250	anti-human IgA, anti-human IgA1, anti-human IgA2, anti-human IgD, anti-human IgM, anti-human IgG2 anti-human IgG3, anti-human IgG4, FcαR
